# Genome Sequence of “*Candidatus* Rickettsia colombianensi,” a Novel Tick-Associated Bacterium Distributed in Colombia

**DOI:** 10.1128/MRA.01433-18

**Published:** 2019-04-04

**Authors:** Jorge Miranda, Salim Mattar, Andrés Puerta-González, Carlos Muskus, José A. Oteo

**Affiliations:** a Instituto de Investigaciones Biológicas del Trópico, Universidad de Córdoba, Montería, Córdoba, Colombia; b Universidad de Antioquia, Medellín, Antioquia, Colombia; c Programa de Estudio y Control de Enfermedades Tropicales (PECET), Facultad de Medicina, Universidad de Antioquia, Medellín, Colombia; d Infectious Diseases Area, Hospital Universitario San Pedro-CIBIR, Logroño, La Rioja, Spain; University of Arizona

## Abstract

This is the first report of the genome sequence of “*Candidatus* Rickettsia colombianensi” strain Adcor 2, deposited in DDBJ/ENA/GenBank under the accession number RAQN00000000. The draft genome showed 36.01% similarity with that of Rickettsia monacensis strain IrR/Munich (NCBI accession number LN794217), 37.81% similarity with that of Rickettsia heilongjiangensis 054 (NCBI accession number CP002912), and 43.88% similarity with that of Rickettsia tamurae AT-1 (NCBI accession number CCMG01000001).

## ANNOUNCEMENT

The genus *Rickettsia* comprises Gram-negative obligate intracellular bacteria belonging to the alphaproteobacteria (1). To date, 32 *Rickettsia* species have been officially validated (http://www.bacterio.net/-allnamesmr.html). About 20 of them are pathogenic, including the agent of Rocky Mountain spotted fever (Rickettsia rickettsii), Mediterranean spotted fever (Rickettsia conorii), and epidemic typhus (Rickettsia prowazekii) (2). About 60 rickettsiae are not validated species and are considered nonpathogenic. Among these rickettsiae, 15 are potential new species classified as having *Candidatus* sp. status (3) (http://www.bacterio.net/-candidatus.html).

“*Candidatus* Rickettsia colombianensi” was described as a *Rickettsia* strain in Amblyomma dissimile ticks removed from iguanas in the municipality of Monteria (Colombia) in 2012. The sequences of the citrate synthase gene (*gltA*), outer membrane protein gene B (*ompB*), and outer membrane protein gene A (*ompA*) were deposited in GenBank under accession numbers JF905456, JF905457, and JF905458, respectively (4). However, “*Ca*. Rickettsia colombianensi” has been detected in different ticks in Colombia (5–7) and several countries in Latin America, such as Brazil (8) and Honduras (9). Although its pathogenesis in vertebrate hosts is unknown, “*Ca.* Rickettsia colombianensi” are in the spotted fever group and cause a strong cytopathic effect in Vero cells.

“*Ca*. Rickettsia colombianensi” was initially isolated from A. dissimile ticks using the shell vial technique (10). Later, for propagation, the isolates were cultivated on Vero cells supplemented with Dulbecco’s modified Eagle’s medium (DMEM) enriched with 5% of fetal bovine serum with iron, without antibiotics. The optimum growth temperature was 28°C with 5% CO_2_. After 7 days of incubation, the level of infection in cells was greater than 90%, a partial purification was performed with a sonication process and centrifugation steps (11). Genomic DNA was extracted with a QIAamp DNA minikit (Qiagen, Valencia, CA), eluted in a final volume of 100 µl, and stored at −20°C.

Genomic DNA was sequenced with PacBio RS II technology, and a library for the PacBio single-molecule real-time (SMRT) sequencing system was processed with P6v2 polymerase binding and C4 chemistry (P6-C4) kits. The total numbers of reads sequenced was 1,550,983,152, and the average read size was 14,917. Raw reads were assembled with PacBio SMRT Analysis pipeline v5.1.0 and the HGAP 4 protocol (12). The default settings for genome assembly were used. The annotation of each of the contigs was made with two strategies: the first was with the RAST tool kit (RASTtk) integrated in PATRIC v.3.5.19 (https://www.patricbrc.org) (13), and the second was with Prokka v1.12 (14). The draft genome of “*Ca*. Rickettsia colombianensi” consists of 13 contigs, with a GC content of 32.46% and an *N*_50_ value of 127,493 bp. The maximum contig length was 177,017 bp. The predicted number of coding sequences (CDSs) is 1,038 with PATRIC and 1,190 with Prokka. However, for the public version of the genome, the RAST tool kit (RASTtk) integrated in PATRIC v.3.5.19 will be used. Furthermore, 18 tRNAs and one transfer-messenger RNA (tmRNA) were identified with Prokka.

This is the first report of the genome sequence of “*Ca*. Rickettsia colombianensi”. The genes of three previously annotated genomes were subjected to a BLAST search against the genome of our *Rickettsia* assembled genome. “*Ca*. Rickettsia colombianensi” strain Adcor 2 showed 36.01% of gene similarity with that of Rickettsia monacensis strain IrR/Munich (NCBI accession number LN794217), 37.81% gene similarity with that of Rickettsia heilongjiangensis 054 (NCBI accession number CP002912), and 43.88% gene similarity with that of Rickettsia tamurae AT-1 (NCBI accession number CCMG01000001).

### Data availability.

The genome sequences have been deposited in DDBJ/EMBL/GenBank under accession number RAQN00000000, BioProject accession number PRJNA493498, and BioSample number SAMN10135122.
